# scMRI Reveals Large-Scale Brain Network Abnormalities in Autism

**DOI:** 10.1371/journal.pone.0049172

**Published:** 2012-11-21

**Authors:** Brandon A. Zielinski, Jeffrey S. Anderson, Alyson L. Froehlich, Molly B. D. Prigge, Jared A. Nielsen, Jason R. Cooperrider, Annahir N. Cariello, P. Thomas Fletcher, Andrew L. Alexander, Nicholas Lange, Erin D. Bigler, Janet E. Lainhart

**Affiliations:** 1 Departments of Pediatrics and Neurology, University of Utah, Salt Lake City, Utah, United States of America; 2 Department of Neuroradiology, University of Utah, Salt Lake City, Utah, United States of America; 3 Interdepartmental Program in Neuroscience, University of Utah, Salt Lake City, Utah, United States of America; 4 The Brain Institute at the University of Utah, Salt Lake City, Utah, United States of America; 5 Department of Psychiatry, School of Medicine, University of Utah, Salt Lake City, Utah, United States of America; 6 School of Computing, University of Utah, Salt Lake City, Utah, United States of America; 7 Scientific Computing and Imaging Institute, University of Utah, Salt Lake City, Utah, United States of America; 8 Waisman Laboratory for Brain Imaging and Behavior, University of Wisconsin, Madison, Wisconsin, United States of America; 9 Departments of Medical Physics and Psychiatry, University of Wisconsin, Madison, Wisconsin, United States of America; 10 Departments of Psychiatry and Biostatistics, Harvard Medical School, Boston, Massachusetts, United States of America; 11 Neurostatistics Laboratory, McLean Hospital, Belmont, Massachusetts, United States of America; 12 Department of Psychology, Brigham Young University, Provo, Utah, United States of America; 13 Neuroscience Center, Brigham Young University, Provo, Utah, United States of America; Bellvitge Biomedical Research Institute-IDIBELL, Spain

## Abstract

Autism is a complex neurological condition characterized by childhood onset of dysfunction in multiple cognitive domains including socio-emotional function, speech and language, and processing of internally versus externally directed stimuli. Although gross brain anatomic differences in autism are well established, recent studies investigating regional differences in brain structure and function have yielded divergent and seemingly contradictory results. How regional abnormalities relate to the autistic phenotype remains unclear. We hypothesized that autism exhibits distinct perturbations in network-level brain architecture, and that cognitive dysfunction may be reflected by abnormal network structure. Network-level anatomic abnormalities in autism have not been previously described. We used structural covariance MRI to investigate network-level differences in gray matter structure within two large-scale networks strongly implicated in autism, the salience network and the default mode network, in autistic subjects and age-, gender-, and IQ-matched controls. We report specific perturbations in brain network architecture in the salience and default-mode networks consistent with clinical manifestations of autism. Extent and distribution of the salience network, involved in social-emotional regulation of environmental stimuli, is restricted in autism. In contrast, posterior elements of the default mode network have increased spatial distribution, suggesting a ‘posteriorization’ of this network. These findings are consistent with a network-based model of autism, and suggest a unifying interpretation of previous work. Moreover, we provide evidence of specific abnormalities in brain network architecture underlying autism that are quantifiable using standard clinical MRI.

## Introduction

The neurobiology of autism has been studied since the initial description of the disorder [1]. Both genetic and non-genetic contributing factors have been implicated, but specific etiologic factors remain undefined. Differences in gross brain morphology, such as early brain overgrowth, have been well documented [2]. More recent studies investigating the nature of these gross abnormalities have produced incongruous results [3]. Frontal lobe volume appears decreased in autism [4], and decreased gray matter (GM) volume in orbitofrontal cortex [5], as well as abnormally thin frontotemporal cortex [6] has been reported. In contrast, others have reported that GM volume and thickness is enlarged in these cortical regions [7]–[9]. Increased GM volume has also been shown in regions involved in social and communicative function including dorsal and medial prefrontal regions, lateral and medial temporal areas, parietal regions, and auditory and visual association cortices [10], [11] [12], [13]. Similarly, discrepant white matter (WM) abnormalities have been reported in autism, including regional increases [14]–[16], as well as decreases in cross-sectional area and microstructure of the corpus callosum [17], [18]. Concomitant white matter disruptions have been reported in prefrontal, superior temporal, temporoparietal cortices, and corpus callosum [19], but increases in whole brain white matter volume have also been observed [8], [16]. How these varied anatomic abnormalities relate to domain-specific cognitive impairment in social and emotional functioning, language and communication deficits, and poor executive function remains unclear.

Functional connectivity MRI (fcMRI) has begun to reveal distinct abnormalities in autism. fcMRI depicts synchronous blood-oxygen-level-dependent (BOLD) signal covariance of spatially discrete brain regions, implying ‘connectedness’ of correlated regions [20]. A consistent finding in fcMRI studies of autism is ‘long range’ underconnectivity, particularly between frontal and temporal cortices and between both of these areas with other brain regions [2], [21]–[24]. Similar to volumetric and DTI studies, however, numerous studies have reported discordant findings [25]. For example, the underconnectivity hypothesis of autism has recently been challenged by work documenting increased connectivity between brain regions, particularly within parietal, temporal, and mesial temporal regions [26]–[29]. Increased local connectivity in frontal regions has also been reported [30], and abnormal recruitment of dorsal frontal regions may occur [31], suggesting that decreased and increased connectivity may coexist [32].

Although inconsistent reports of over- versus under-growth and connectivity may result from technical or methodological differences [25], [26], these findings could reflect discrete abnormalities in brain network neurobiology. Conserved topological distribution of fcMRI signal covariance has revealed a finite set of canonical domain-specific ‘resting state’ or ‘intrinsic connectivity’ networks (ICNs) in the human brain [33]–[35]. Many brain regions with reported abnormalities in autism, including those exhibiting weaker connectivity [22], , in addition to those with stronger connectivity [29], [36], lie within canonical ICNs. Numerous groups have now reported altered fMRI connectivity in specific brain regions that appear to lie within ICNs, including the salience network (SN) and the default mode network (DMN) [37], [38]. Similarly, volumetric and DTI studies often report regional assessment of areas that comprise ICN architecture. Network-specific abnormalities could account for discrepant volumetric, DTI, and fcMRI findings, providing a unifying framework for interpreting these data. Regionally selective abnormalities would be expected to produce differential brain structure in specific brain regions within a canonical large-scale network, whereas closely approximated regions outside of the network may not be affected. Moreover, across networks, some brain regions may demonstrate increased growth or connectivity, whereas others may display underdevelopment or underconnectivity. Under a network model of autism, brain volume may be preserved, but maldeveloped network architecture could result in domain-specific abnormalities that characterize the disease.

Emerging evidence supports abnormal connectivity of subregions within the DMN in autism. This network, comprised of medial and ventral prefrontal cortex, retrosplenial cortex, bilateral angular gyrus, and anchored by posterior cingulate cortex (PCC) is active during “rest”, in the absence of goal-oriented or externally-directed stimuli, and normally deactivates during performance of cognitively demanding tasks [39]–[41]. The DMN is thought to mediate internally-directed processes [42], and is involved in detection of and directing attention to mental events during the resting state [43], processes which represent abnormal clinical traits seen in autism [44]. Multiple studies have documented fcMRI abnormalities in brain regions within this network [26], [37], [38], [45].

Social functioning relies upon frontoinsula, anterior cingulate, amygdala, fusiform gyrus, and ventral prefrontal cortex [46]–[48], and fcMRI abnormalities within many of these areas have been demonstrated in autism [46], [47]. These regions overlap extensively with a canonical ICN known as the salience network (SN) [48]. The SN, comprised of frontoinsular, dorsal anterior cingulate, and subcortical structures including amygdala, substantia nigra, and ventral tegmental area, integrates conflict monitoring, autonomic responses, and reward processing so that an organism can decide what to do (or not to do) next [48]. The integration of external stimuli with internal state maintains homeostasis in a dynamic environment comprised of nearly continuous decision-making in the awake, alert state [47], [49]. The main hub of the salience network is the anterior frontoinsula (FI), a region that has been reported to be hypoactive in functional MRI studies of autism [50]–[52] and undergoes early degeneration in other conditions characterized by social-emotional dysfunction such as frontotemporal dementia [53], [54].

In addition, the anterior frontoinsula is uniquely positioned to mediate interactions between two counterposed large-scale networks, the DMN and the executive-control network (ECN) [47], [55]. This switch dynamically evaluates sensory stimuli and subsequently engages internal (DMN) versus external (ECN) processing streams [47], [56], directing DMN and ECN dominance and subsequent downstream information processing [47], [55], [56]. The salience network thus may serve dual critical functions in autism, first evaluating salient socio-emotional stimuli, and second mediating interactions between large-scale networks involving external- and self-directed processes via its hub, the FI [47], [56].

Despite considerable efforts delineating regional abnormalities in autism, little is known regarding large-scale network-level architecture that may underlie the cognitive and behavioral manifestations of the disease. Numerous studies report functional and structural abnormalities in regions containing canonical network nodes, suggesting collectively that network-level abnormalities may be a fundamental characteristic of autistic neurobiology. The most prominent clinical features, namely marked deficits in social and emotional functioning as well as dysregulation of internally- versus externally-directed behaviors, predict network-level abnormalities in the SN and DMN respectively. We examined whether structural abnormalities in large-scale distributed brain networks could be detected in autism, and hypothesized that autism would demonstrate altered network-level structural architecture consistent with poor development or restricted topology of the SN, as well as overgrowth of the DMN. We employed structural covariance MRI (scMRI) techniques [54], [57], [58] to determine network-specific topology of whole-brain GM signal covariance. scMRI reveals brain regions with co-varying GM density across subjects, suggesting shared developmental or genetic influences. Using this approach, we examined whether network-level structural brain architecture is abnormal in autism, whether reported functional abnormalities may have a structural basis, and whether structural network abnormalities are consistent with clinical aspects of the disease.

## Materials and Methods

### Subjects and MRI Methods

Forty-nine male subjects with autism aged 3–22 years were compared to forty-nine age- and IQ-matched normal male control subjects. Diagnosis of autism was established using the Autism Diagnostic Interview-Revised (ADI-R) [59], Autism Diagnostic Observation Schedule-Generic (ADOS-G) [60], DSM-IV (American Psychological Association, 1994) and ICD-10 criteria by a trained expert (JEL). Individuals with comorbid neurogenetic conditions (Fragile-X, abnormal karyotype, characteristic history or physical examination findings) were excluded. Control subjects also were assessed with the ADOS-G [60] to confirm typical development. Group characteristics are shown in Table 1. Age distribution is shown in Table S1. Images were acquired using a Siemens 3.0 Tesla Trio MRI scanner. Whole brain isotropic MPRAGE image volumes were acquired in the sagittal plane using an 8-channel receive-only RF head coil, employing standard techniques (TR = 2300 ms, TE median = 3 ms, matrix median = 256×256×160, flip angle 12°, voxel resolution = 1 mm^3^, acquisition time = 9 min 12 sec). Images were inspected for motion artifact at the time of scan and reacquired if considered of low quality. During post-processing, each image volume was inspected visually both pre- and post-segmentation, to ensure image quality and accurate tissue classification. Image volumes were discarded if motion aliasing was detected or resulted in image degradation or segmentation failure.

**Table 1:** Group demographics and neuropsychiatric test characteristics.

	Mean Age (yrs.)	S.D. Age (yrs.)	Age Range (yrs.)	ADOS-SI	ADOS-C	ADOS tot	VIQ	PIQ
*autism*	13.27 (5.07)	5.07	3.49–22.33	9.47	4.9	14.37	98.15	95.55
*control*	13.67 (5.53)	5.53	3.47–22.44	0.72	0.49	1.21	110.7	106.77

ADOS-SI, ADOS Social Impairment score; ADOS-C, ADOS Communication score; VIQ, Verbal IQ; PIQ, Performance IQ.

### Data Analysis

We created customized image analysis templates by normalizing, segmenting, and averaging MPRAGE images using SPM5 (http://www.fil.ion.ucl.ac.uk/spm) according to a recently proposed processing pipeline [61], [62]. First, images were transformed into standard space using a 12-parameter affine-only linear transformation, and segmented into three tissue classes representing gray matter, white matter, and cerebrospinal fluid. Smoothly varying intensity changes as well as artifactual intensity alterations as a result of the normalization step were corrected for using a standard modulation algorithm within SPM5. The resulting segmented volumes were then smoothed using a 12 mm full-width at half-maximum Gaussian kernel. To study network structural covariance, we derived gray matter intensities using 4 mm radius spherical seed regions of interest (ROIs) chosen using previously published ICN peak foci from the literature and adjusted to our childhood template using relevant anatomical landmarks to avoid seed placement too near the pial surface. Seed ROIs were selected within right frontoinsular cortex [48] and right posterior cingulate cortex [63]. These regions anchor the salience and default mode networks, respectively [40], [48]. We performed condition-by-covariate analyses for each seed region in which the mean seed gray matter intensity was the covariate of interest and disease status was the grouping variable. Total brain volume (TBV) was included as a covariate-of-no-interest. This design enabled us to determine the whole-brain patterns of seed-based structural covariance in each group. One-sample t-tests were performed to reveal voxels with significant groupwise gray matter density covariance across subjects. Resulting statistical parametric maps were thresholded at p<0.01 corrected for family-wise error (FWE) and displayed on the normalized brain template to allow qualitative comparisons of network topology between groups. To quantify differences in network volume, we calculated the total number of significant voxels in each network and plotted these by group. Between-group contrasts sought regional head-to-head differences in seed covariance between groups (p<0.05, inclusively masked to the network global map for both groups at p<0.01 FWE). Peak voxel and cluster characteristics were generated using the aal toolbox within SPM5 (http://www.cyceron.fr/web/aal__anatomical_automatic_labeling.html). To determine whether ADOS Social Impairment scores were related to differential brain structure in autism, we performed scMRI using ADOS-SI scores as the covariate of interest. Between-group analysis reveals group differences in covariance patterns, independent of seed volume or network constraints.

Age effects were modeled as covariates-of-no-interest but resulted in minimal appreciable topological differences in the resulting maps, likely due to sample size, group matching, and statistical constraints of the scMRI technique (see Fig. S1, Table S2, Table S3).

### Ethics

This research was approved and conducted according to the policies, procedures, and regulatory oversight of the University of Utah IRB. All consent, scanning, data management, and analytical procedures were performed in accordance with these policies and procedures. Written informed consent and/or assent was obtained for each participant and/or guardian according to IRB-approved procedures. Capacity to consent was assessed by a trained study staff member present at time of consent. Guardians were asked to give consent on behalf of participants deemed unable or<18 years of age. Signed consent documentation is maintained in the laboratory and a copy was provided to the patient and/or guardian. The University of Utah IRB complies with all U.S regulatory requirements related to the protection of human subjects research participants.

## Results

**Figure 1:**
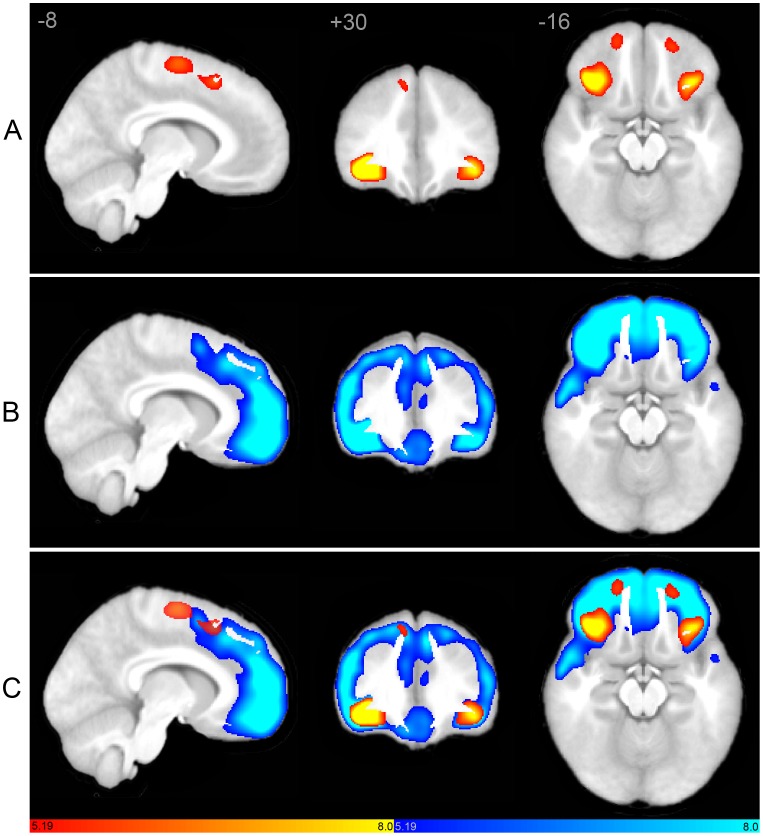
Structural covariance maps of the salience network in autism and controls. Statistical parametric maps depict brain regions in which gray matter intensity covaried with that of the seed ROI (right FI) in each group. (A) Structural covariance patterns appear substantially spatially restricted in autism (hot colors; see also Table 2). (B) Corresponding scMRI map in normal controls corresponds to a robust canonical SN (cool colors). (C) scMRI maps from both groups overlaid on a single anatomic volume. scMRI data are T-statistic maps (p<0.01, FWE-corrected) displayed on the average anatomical template of all subjects. The left side of the image corresponds to the right side of the brain. FI, frontoinsula; FWE, family-wise error; ROI, region of interest; scMRI, structural covariance MRI; SN, salience network.

**Figure 2:**
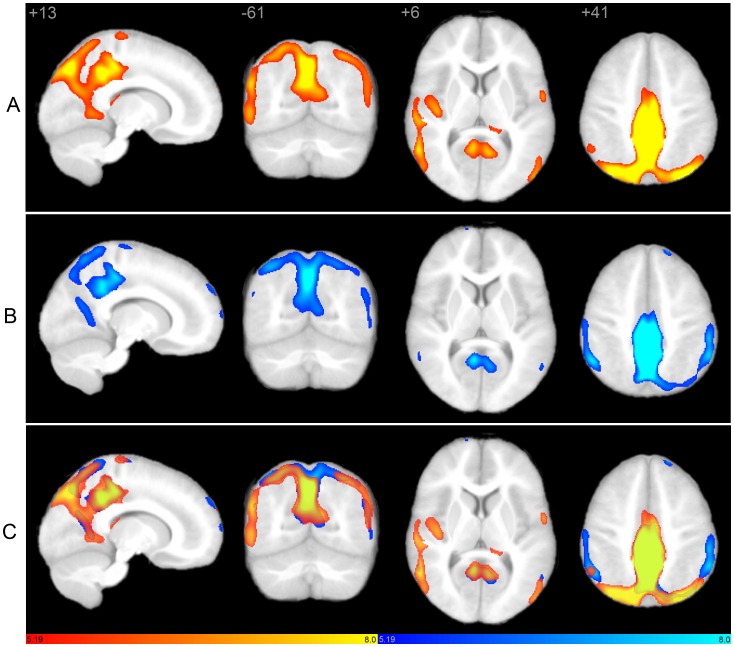
Structural covariance maps of the default mode network in autism and controls. Statistical parametric maps depict brain regions in which gray matter intensity covaried with that of the seed ROI (right PCC) in each group. (A) Structural covariance patterns appear robust in posterior brain regions, but restricted in frontal areas in autism (hot colors; see also Table 2). Covariance outside of canonical DMN boundaries is also evidenced. (B) Corresponding scMRI map in normal controls corresponds to a robust canonical default mode network (cool colors). (C) scMRI maps from both groups overlaid on a single anatomic volume. scMRI data are T-statistic maps (p<0.01, FWE-corrected) displayed on the average anatomical template of all subjects. The left side of the image corresponds to the right side of the brain. DMN, default mode network; FWE, family-wise error; PCC, posterior cingulate cortex; ROI, region of interest; scMRI, structural covariance MRI.

Seed-based scMRI revealed expected structural covariance maps in normal controls [58], consistent with canonical structural covariance network (SCN) and ICN topologies. However, marked differences were observed in both SN and DMN in autistic subjects. As predicted by clinical phenotype, the pattern of abnormality was divergent in these networks. The SN demonstrated restricted extent and distribution (Figure 1). In contrast, the DMN showed overgrowth in discrete posterior regions, in tandem with decreased nodal covariance in frontal regions (Figure 2).

### Salience Network

**Figure 3:**
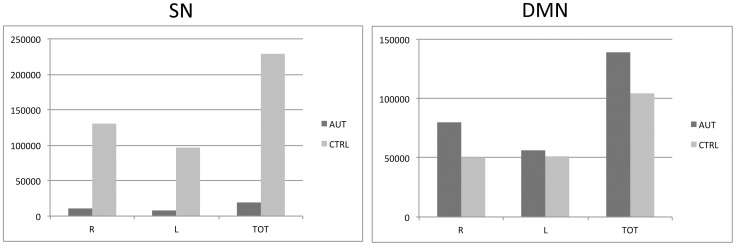
Group differences in network volume. Plots of voxel counts by group indicate substantially restricted network extent in the SN of autistic subjects, whereas the DMN is more spatially extensive in the autistic group. Y-axis scale is voxel number from associated statistical maps. AUT, autism group; CTRL, control group; DMN, default mode network; L, left; R, right; SN, salience network.

In our control group, the right FI anchored covariance maps (Figure 1) that included extensive medial and lateral frontal regions, including most of the medial frontal wall as well as bilateral frontoinsular, orbitofrontal, dorsal anterior cingulate, frontopolar, and anterior temporal cortex, recapitulating canonical fcMRI and scMRI SN topology [48], [58]. In contrast, autistic subjects demonstrated less robust covariance with right FI. SN volume was 8% of normal in overall volume (Figure 3), with restricted topological distribution versus controls. Covariance was undetected in autism in most of the hallmark regions of the SN, and was limited to homologous left FI and discrete nodes in bilateral orbitofrontal and dorsal cingulate regions, as well as right superior frontal gyrus and left supplementary motor cortex. Medial frontal covariance included nodes in the autistic group that extended more posteriorly versus the control group. Peak clusters and coordinates are depicted in Table 2.

### Default Mode Network

In controls, the right PCC seed covaried with bilateral precuneus/retrosplenial cortex, lateral parietal regions including angular gyrus, right medial prefrontal cortex, and discrete nodes within right superior frontal gyrus and medial frontal cortex (Figure 2), corresponding to canonical DMN topology [39]–[41]. Robust canonical DMN frontal and mesial temporal covariance was not observed in the control group, confirming previous reports of immature DMN topology in children of similar age as our sample average [58], [63]. In autistic subjects, PCC showed widespread signal covariance with posterior nodes in canonical DMN regions, as well as additional regions within medial parietal and occipito-parietal cortex that were not seen in controls. Covariance within prefrontal DMN regions was not evidenced in the autism group. Moreover, discrete nodes outside of canonical DMN boundaries also covaried with PCC in the autism group, including regions commonly associated with autism such as right fusiform, right inferior temporooccipital junction, right middle and superior temporal gyrus, right frontal operculum including inferior frontal gyrus, bilateral caudate body, and left orbitofrontal cortex. Total network volume was increased in autism versus controls (Figure 3). Peak clusters and coordinates are depicted in Table 2.

**Table 2:** MNI coordinates and characteristics of peak voxels and associated clusters of groupwise scMRI maps.

	x	y	z	p (FWE)	T height	Peak Region	Secondary Regions		
**SN (R FI seed)**									
*autism*	38	26	−10	0.000	94.87	R FI			
	−37	27	−12	0.000	9.16	L FI			
	−30	21	−19	0.000	6.68	L FI	L Insula		
	10	−6	49	0.000	6.69	R SMA	R Mid Cingulum		
	10	14	50	0.000	5.99	R SMA	R Sup Frontal		
	10	27	49	0.001	5.9	Medial R Sup Frontal	R SMA	R Sup Frontal	
	−12	−9	60	0.000	6.6	L SMA			
	−7	17	48	0.000	6.15	L SMA	L Sup Frontal		
	−20	8	55	0.000	6.1	L Sup Frontal	L Mid Frontal		
	19	53	−17	0.001	5.86	R Sup Frontal Orb	R Mid Frontal Orb		
	11	55	−24	0.003	5.53	R Sup Frontal Orb	Rectus		
	−21	48	−15	0.001	5.86	L Mid Frontal Orb	L Sup Frontal Orb		
	20	56	1	0.002	5.65	R Sup Frontal	R Sup Frontal Orb	R Med Sup Frontal	
	−49	−1	39	0.004	5.41	L Precentral	L Postcentral		
	7	18	−19	0.006	5.34	R Rectus	R Olfactory		
	−5	23	−23	0.007	5.29	L Rectus	L Sup Frontal Orb		
	−53	−14	23	0.007	5.29	L Postcentral			
	1	14	−1	0.008	5.27	L Caudate	R Caudate	B Olfactory	
	52	6	23	0.008	5.26	R Inf Frontal Oper	R Precentral		
	26	41	41	0.008	5.25	R Sup Frontal	R Mid Frontal		
	11	51	−9	0.008	5.24	R Med Frontal Orb	R Sup Frontal Orb		
	−53	1	11	0.009	5.23	L Rolandic Oper	L Inf Frontal Oper	L Postcentral	
*control*	38	26	−10	0.000	116.57	R FI	R Insula		
	23	59	2	0.000	12.19	R Sup Frontal	R Sup Frontal Orb		
	−24	58	−3	0.000	11.3	L Sup Frontal Orb	L Sup Frontal		
	−37	−28	−27	0.001	5.93	L Fusiform	L Inf Temporal		
	−15	−11	58	0.001	5.74	L SMA			
	−53	10	9	0.005	5.36	L Inf Frontal Oper	L Rolandic Oper		
**DMN (R PCC seed)**									
*autism*									
	4	−40	36	0.000	252.96	R Mid Cingulum	L Mid Cingulum	B Precuneus	
	11	−70	42	0.000	9.81	R Precuneus	R Cuneus		
	−33	−76	37	0.000	8.21	L Mid Occipital	L Inf Parietal		
	−3	−16	−16	0.000	7.79	L Ventral Tegmental			
	−7	−30	12	0.000	7.57	L Thalamus			
	−17	−29	23	0.000	6.27	L Caudate			
	−16	−38	−2	0.009	5.23	L Lingual	L Hippocampus		
	11	−21	−14	0.000	6.64	R Parahippo			
	−53	9	−14	0.000	6.49	L Sup Temporal Pole			
	−19	15	−23	0.000	6.38	L Inf Frontal Orb	L Sup Frontal Orb		
	21	−29	70	0.000	6.28	R Precentral	R Postcentral		
	11	−22	72	0.000	6.16	R Paracentral Lobule	R SMA	R Precentral	
	0	−25	15	0.000	6.25	L Thalamus			
	10	−31	12	0.000	6.06	R Thalamus			
	−58	−2	8	0.000	6.03	L Rolandic Oper	L Sup Temporal	L Postcentral	
	17	−26	23	0.001	5.98	R Caudate			
	17	64	26	0.001	5.77	R Sup Frontal	R Med Sup Frontal		
	−13	−26	−22	0.002	5.7	L Parahippo			
	−58	6	27	0.003	5.57	L Precentral	L Inf Frontal Oper		
	−18	54	37	0.003	5.5	L Sup Frontal			
	−19	63	24	0.005	5.38	L Sup Frontal			
	−52	38	8	0.005	5.38	L Inf Frontal Tri			
	−43	13	0	0.005	5.38	L Insula	L Inf Frontal Oper	L Inf Frontal Tri	
	−20	16	60	0.005	5.38	L Sup Frontal	L Mid Frontal		
	−21	−37	67	0.006	5.36	L Postcentral			
	27	−30	−41	0.006	5.35	cerebellum			
	−30	−29	−33	0.007	5.31	L Fusiform	Cerebellum		
	15	−20	25	0.007	5.3	R Caudate			
	3	34	−33	0.008	5.26	Rectus (bilat)			
	−5	−24	70	0.010	5.21	L paracentral lobule			
*control*	4	−40	36	0.000	227.54	R Mid Cingulum	L Mid Cingulum	B Precuneus	
	−9	−52	64	0.000	7.64	L Precuneus			
	38	−55	51	0.000	7.36	R Inf Parietal	R Sup Parietal	R Angular	
	−18	54	37	0.000	6.14	L Sup Frontal			
	15	59	33	0.001	5.84	R Sup Frontal	R Sup Medial Frontal		
	−57	5	30	0.001	5.74	L Precentral			
	−61	−6	34	0.002	5.59	L postcentral	L Precentral		
	−8	−27	16	0.003	5.54	L Thalamus			
	−26	13	61	0.004	5.47	L Mid Frontal	L Sup Frontal		
	14	70	9	0.005	5.42	R Sup Medial Frontal	R Sup Frontal		
	21	25	55	0.005	5.4	R Sup Frontal			
	21	35	49	0.007	5.3	R Sup Frontal			
	−58	−6	3	0.008	5.27	L Sup Temporal	L Rolandic Oper		
	−7	−23	69	0.008	5.26	L Paracentral Lobule			
	−41	−79	20	0.010	5.21	L Mid Occipital			

FWE, family-wise error.

### Between-group Analyses

**Figure 4:**
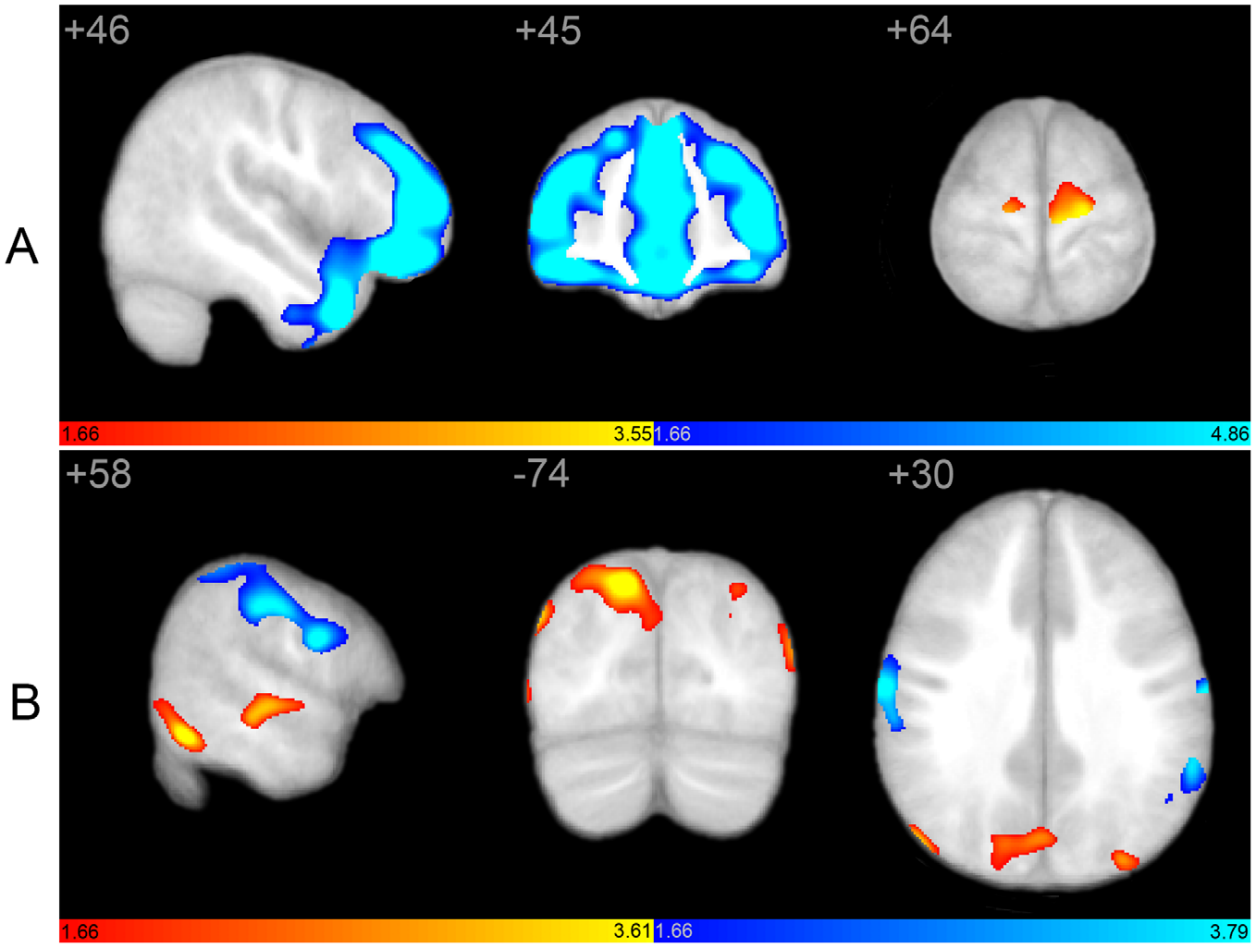
Structural covariance maps of salience and default mode networks as a result of direct between-group contrasts. Statistical parametric maps depict brain regions in which gray matter intensity covaried with that of the seed ROI (right FI or PCC) differently between groups. (A) Structural covariance with right FI is greater in bilateral SMA in autistic subjects (hot colors; see also Table 3), whereas control subjects (cool colors) demonstrate more robust covariance in extensive frontal and temporal brain regions, in addition to insular cortex. Covariance outside of canonical SN boundaries is evidenced only in the autistic group. (B) Structural covariance with right PCC includes posterior cingulate, parieto-occipital, and temporal brain regions in autism (hot colors), whereas frontal covariance is absent. In contrast, control subjects demonstrate more robust covariance in frontal, lateral inferior parietal, and paracentral regions (cool colors; see also Table 3). Covariance outside of canonical DMN boundaries is evidenced only in the autistic group. scMRI data are T-statistic maps (p<0.05, inclusively masked to the network global map for both groups at p<0.01 FWE) displayed on the average anatomical template of all subjects. The left side of the image corresponds to the right side of the brain. DMN, default mode network; FI, frontoinsula; FWE, family-wise error; PCC, posterior cingulate cortex; ROI, region of interest; SN, salience network.

Direct contrasts between autism and control groups revealed characteristic regional differences, confirming the above groupwise analyses (Figure 4). Within the SN, control subjects had greater covariance in extensive bilateral frontal regions, as well as anterior cingulate, anterior temporal cortex, and left insula (Table 3). Autistic subjects had increased covariance in bilateral supplementary motor area (SMA), bilateral caudate, and right insular cortex. Consistent with group analyses, PCC covariance was restricted to posterior brain regions in autistic subjects, and included regions within the canonical DMN such as retrosplenial cortex and bilateral angular gyrus, but also included regions outside of the canonical DMN including medial inferior parietal cortex bilaterally, and right superior, middle, and inferior temporal as well as right Heschl’s gyrus. In controls, PCC covaried with regions comprising the canonical DMN, including anterior as well as posterior nodes. Autistic subjects showed greater right precuneus and medial inferior parietal covariance, whereas control subjects had greater left precuneus and lateral inferior parietal covariance.

**Table 3:** MNI coordinates and characteristics of peak voxels and associated clusters of between-group scMRI maps.

	x	y	z	p (UC)	T height	Peak region	Secondary Regions		
**SN (R FI seed)**									
*autism>control*	−14	−15	62	0.014	3.55	L SMA	L Paracentral Lobule	L Precentral	
	−10	−6	58	0.02	2.07	L SMA	L Paracentral Lobule		
	−20	−6	58	0.027	1.95	L Sup Frontal	L Mid Frontal		
	36	27	−10	0.029	1.92	R Inf Fronal Orb (FI)	R Insula		
	12	−3	55	0.033	1.86	R SMA	R Mid Cingulum		
	−49	1	40	0.036	1.81	L Precentral			
	2	16	−2	0.043	1.73	R olfactory	R Caudate	L Caudate	L Olfactory
*control>autism*	42	39	−14	0	4.86	R Mid Frontal Orb			
	−28	55	7	0	4.2	L Mid Frontal	L Sup Frontal		
	27	55	12	0	4.03	R Sup Frontal	R Mid Frontal		
	−7	51	14	0	3.61	L Medial Sup Frontal	L Ant Cingulate		
	−11	56	5	0	2.87	L Medial Sup Frontal	L Sup Frontal		
	−5	30	40	0	2.36	L Medial Sup Frontal			
	45	9	−32	0	3.49	R Mid Temporal Pole	R Sup Temporal Pole		
	−37	46	17	0.001	3.19	L Mid Frontal	L Inf Frontal Tri		
	−31	44	26	0.001	3.05	L Mid Frontal			
	−18	47	34	0.013	2.24	L Sup Frontal	L Mid Frontal		
	−24	41	35	0.013	2.09	L Sup Frontal	L Mid Frontal		
	16	54	18	0.017	2.16	R Sup Frontal	R Medial Sup Frontal		
	−49	32	18	0.036	1.81	L Inf Frontal Tri			
	−43	11	−26	0.041	1.74	L Sup Temporal Pole	L Mid Temporal Pole		
	−40	12	−29	0.046	1.69	L Mid Temporal Pole	L Sup Temporal Pole		
	−44	27	−15	0.047	1.68	L Inf Frontal Orb (FI)	L Sup Temporal Pole		
	51	23	−2	0.047	1.68	R Inf Frontal Tri	R Inf Frontal Orb (FI)		
	−43	24	−15	0.049	1.66	L Inf Frontal Orb (FI)	L Sup Temporal Pole		
**DMN (R PCC seed)**									
*autism>control*	14	−73	40	0.003	3.61	R Precuneus	R cuneus		
	2	−70	30	0.019	3.58	R Precuneus	L cuneus	L Precuneus	R cuneus
	−30	−78	35	0.01	3.51	L Mid occipital	L Inf parietal		
	46	−74	28	0.011	3.48	R Mid Occipital	R Angular		
	52	−66	25	0.017	2.86	R Angular	R Mid Temporal	R Mid Occipital	
	57	−24	−3	0.017	2.15	R Sup Temporal	R Mid Temporal		
	59	−54	−11	0.024	2	R Inf Temporal	R Mid Temporal		
	57	−58	−3	0.03	1.9	R Mid Temporal	R Inf temporal		
	−48	−76	16	0.025	1.98	L Mid Occipital	L Mid Temporal		
	48	−13	1	0.044	1.71	R Sup Temporal	R Insula	R Heschl	
	53	−61	15	0.048	1.67	R Mid Temporal			
*control>autism*	−56	−46	39	0.003	3.79	L Inf Parietal	L Supramarginal		
	−49	−47	46	0.017	3.59	L Inf Parietal			
	−46	−34	49	0.018	3.51	L postcentral	L Inf Parietal		
	61	1	24	0.004	3.5	R precentral	R postecentral		
	49	−36	53	0.007	2.5	R Inf Parietal	R postcentral		
	50	−27	53	0.017	2.16	R postcentral			
	−6	−58	57	0.009	2.42	L precuneus			
	−19	−56	54	0.015	2.19	L Sup Parietal	L precuneus		
	−11	−50	53	0.028	1.93	L Precuneus			
	−51	−30	48	0.012	2.3	L Inf Parietal	L postcentral		
	−57	−56	−6	0.018	2.12	L mid temporal	L Inf temporal		
	62	−6	18	0.019	2.09	R postcentral	R Rolandic Oper		
	−62	−8	33	0.029	1.91	L postcentral			
	0	−50	−2	0.031	1.88	Vermis	L Cerebellum		
	−26	−32	60	0.033	1.85	L postcentral	L precentral		
	8	−16	58	0.036	1.81	R SMA			
	13	70	8	0.039	1.77	R Medial Sup Frontal	R Sup Frontal		
	21	23	56	0.039	1.77	R Sup Frontal			
	62	−19	18	0.041	1.75	R Supramarginal	R Rolandic Oper	R Postcentral	R Sup Temporal
	−49	−56	26	0.043	1.73	L Angular	L Mid Temporal	L Supramarginal	
	−28	12	61	0.045	1.7	L Mid Frontal			
	62	−15	19	0.047	1.68	R Postcentral	R Rolandic Oper	R Supramarginal	
	−29	15	60	0.047	1.68	L Mid Frontal			
	20	27	54	0.048	1.67	R Sup Frontal			
	19	26	55	0.049	1.66	R Sup Frontal			

UC, uncorrected.

### ADOS-SI Covariance

**Figure 5:**
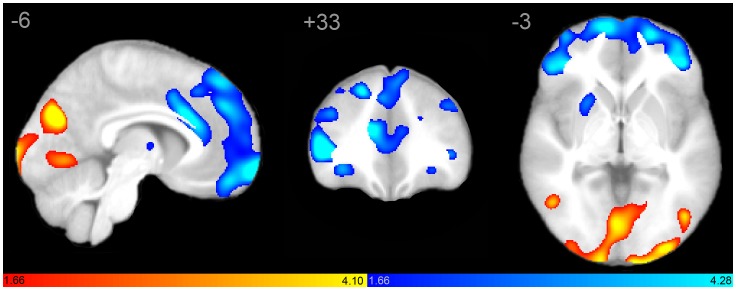
Whole brain structural covariance with ADOS-SI scores as a result of direct between-group contrasts. Statistical parametric maps depict brain regions in which gray matter intensity covaried with ADOS Social Impairment (ADOS-SI) score differently between groups. In controls, ADOS-SI scores covaried with frontal regions overlapping with SN including medial frontal wall, anterior cingulate, and frontoinsular cortex. In contrast, in autistic subjects ADOS-SI covaried with posterior brain regions including cuneus, precuneus, parieto-occipital regions, and temporoparietal cortex. scMRI data are T-statistic maps (p<0.05, inclusively masked to the network global map for both groups at p<0.01 FWE) displayed on the average anatomical template of all subjects. scMRI maps from both groups overlaid on a single anatomic volume reveal distinct between-group differences. The left side of the image corresponds to the right side of the brain. SN, salience network.

We hypothesized that the marked abnormalities in SN architecture could result in poor social and emotional function, which in turn may be reflected in ADOS Social Impairment scores. To determine whether ADOS Social Impairment (ADOS-SI) scores were related to differential brain structure in autism, we analyzed whole-brain GM covariance using ADOS-SI scores as the covariate of interest. Direct contrasts between autism and control groups revealed distinct differences in structural architecture (Figure 5). In autistic subjects, ADOS-SI covaried with posterior brain regions including cuneus, precuneus, parieto-occipital regions, and temporoparietal cortex. Many of these regions overlap with PCC covariance seen in the abnormal autistic DMN (see Fig. 2). In contrast, ADOS-SI scores in the control group covaried with frontal regions overlapping with canonical SN regions (see Fig. 1) including medial frontal wall, anterior cingulate, and frontoinsular cortex.

## Discussion

We demonstrate that specific abnormalities in brain network structure are present in autism. Moreover, the topology of network-level abnormalities is consistent with prior morphological and functional work as well as clinical hallmarks of the disease. Specifically, the SN, anchored by the right FI, appears underdeveloped in autism. In addition, distinct nodal differences are apparent. Frontal regions are underrepresented in autism, whereas SMA covariance exceeds that of controls. In contrast, the DMN, anchored by PCC, may have both ‘overconnected’ and ‘underconnected’ components. Covariance beyond normal DMN topology was evidenced in autism, and this overrepresentation of the DMN was restricted to posterior brain regions. Many of the regions outside of DMN boundaries have been previously implicated in autism, including caudate, inferotemporal/fusiform cortex, auditory and temporal association cortex, and lateral postero-occipital regions. In contrast, covariance with frontal canonical DMN nodes was nearly absent in autism. These data suggest that distinct and network-specific alterations in structural architecture may underlie autism, providing a plausible neural substrate for clinical hallmarks of the disease. Furthermore, these data suggest an anatomic substrate for abnormal functional connectivity reported in autism, and demonstrate that this abnormal architecture is observable using standard anatomic MRI.

Our findings provide a resolution for previously reported inconsistencies in both structural and functional brain architecture in autism, as network-level effects could drive these apparent differences. Regional variability in GM and WM volume, WM integrity, cortical thickness, functional connectivity, and microscopic structure are predicted by network-level abnormalities. A framework of altered network-specific architecture unifies earlier divergent reports suggesting under- and overconnectivity within the same gross brain regions in autism and remains consistent with earlier hypotheses of abnormal connectivity [64], [65]. Similarly, previous reports of gross brain overgrowth can be interpreted in the context of selective network overdevelopment, as the gross anatomic overgrowth that has long characterized the disease may result from network-specific overgrowth or overconnectivity. The plausibility of coexisting over- and underconnectivity in autism is supported by recent fcMRI and structural studies. Noonan et al. [27] reported overconnectivity in posterior regions including visual association and posterior inferotemporal cortex, but underconnectivity in prefrontal regions. Mengotti et al. [66] described regional increased gray matter volumes in posterior regions of autistic subjects, as well as decreased gray matter volumes in frontal regions. Our findings are consistent with reports of both broad-based [21], [67] as well as selective [26], [44], [45] connectivity abnormalities, as a network-level framework predicts widespread but specific alterations in connectivity.

Our data suggest restricted topology of the SN in autism. These results support the notion that autistic subjects lack the cognitive mechanism to disambiguate relevant social and emotional cues within complex environmental stimulus streams [44]. Further, autistic individuals may not possess a robust neuroanatomic substrate for appropriate socio-emotional cognitive processing. Numerous reports suggest that DMN and ECN are functionally anti-correlated in healthy individuals, and their dynamic balance is modulated by the salience network [47], [55], [56]. In this context, our data may reflect a structural basis for an as yet unreported functional imbalance between the DMN and ECN in autism.

Posterior overgrowth and ‘anterior-posterior disconnection’ of the autistic DMN may reflect a perturbed ‘division of labor’ within this network. Our data support an emerging theme in the literature of anterior-posterior underconnectivity [68], but also suggest coexisting specific regional overconnectivity within and outside of the DMN. However, as frontal structural covariance in canonical DMN nodes was modest in our control group, the finding of decreased frontal covariance in autism needs to be confirmed in older subjects, when a mature DMN is expected. However, medial prefrontal cortex has been shown previously to have decreased functional connectivity with other regions in the DMN [26], [37], [69], particularly with posterior nodes [23], [36], [67]. This anterior-posterior disconnection is underscored by overconnectivity within posterior nodes themselves [26], [27], [70]. Using a PCC seed, Monk et al. [26] showed decreased anterior-posterior connectivity *and* increased connectivity between PCC and other posterior regions, precisely consistent with our results using the same seed. Weaker connectivity between select DMN nodes such as prefrontal cortex and angular gyrus has also been shown [37]. Moreover, posterior DMN connectivity was present in autistic subjects, but anterior nodes of the DMN were not apparent. Consistent with our results, they also detected connectivity with nodes outside of the canonical DMN. Kennedy et al. [42] demonstrated that the DMN remains abnormally engaged during resting conditions in autism, and reported subnormal activity in medial orbitofrontal cortex with emotional stimuli. Weng et al. [45] reported that poorer verbal and non-verbal communication scores in autistics correlated with stronger connectivity versus controls between PCC and bilateral temporal cortex including regions that overlap with our autistic DMN scMRI map. Social impairment in autism is associated with decreased connectivity between PCC and superior frontal as well as medial prefrontal regions [26], [45], consistent with our results and plausibly relating to the dual function of FI in autism.

The neural systems that support social and emotional processing appear to be underdeveloped in autism, whereas those that mediate internally- versus externally-directed processing appear to be overrepresented in posterior nodes but isolated from anterior network nodes. The neuropathological processes underlying these selective abnormalities in large-scale brain networks in autism remain unknown. Plausible factors include early neuronal excess and later neuronal loss, abnormal microstructure, excess synapse formation, excessive dentritic outgrowth or hyperconnectivity, aberrant axonal pathfinding, overgrowth, or connections, and altered myelination [71]–[77]. Each of these processes may be driven by aberrant gene expression, environmental factors, or both, and may progress by age-, network-, or domain-specific mechanisms.

How these processes relate to gray matter density is unclear. Our work suggests, however, that downstream effects of presumably disrupted molecular and cellular mechanics produce distinct and measurable alterations of normal brain network architecture within networks that underlie the core manifestations of the autistic disease state. Posterior DMN subnets may be overconnected, whereas rostro-caudal connectivity may be limited. In the SN, interconnections between critical nodes may be malformed, mature architecture not achieved, and the network left rudimentary and dysfunctional. Phenotypic features of autism could result as network ‘dysconnection’ leads to a deficit of ‘salience filtering’ and resultant inefficiencies in recruiting appropriate attentional, socio-emotional, behavioral, and higher-order cognitive resources [47]. This could impact cognitive and behavioral functioning by at least three mechanisms. First, integration of external sensory information with visceral, autonomic, and hedonic status is aberrant, yielding breakdown of appropriate behavioral guidance from relevant internal and external stimuli. Second, social and emotional cues are not properly input to downstream processing pathways, resulting in misguided response-selection. Third, the modulator of externally- versus internally-directed stimulus processing pathways is ‘dysconnected’, resulting in inappropriate signal filtering and generating abnormal engagement of downstream cognitive processing streams and dysfunctional disengagement of the DMN.

We describe anatomic substrates consistent with altered functional connectivity reported in autism, and demonstrate that structural network-level abnormalities are quantifiable using standard anatomic MRI. It is plausible that multiple large-scale network architectures, including ICNs and SCNs, may be affected in autism. Our work predicts abnormal fcMRI covariance within large-scale networks commensurate with specific structural abnormalities that together disrupt emergence and maintenance of complex psychological and physiological functions in autism. However, although connectivity is often assumed by techniques measuring MRI signal covariance, neither fcMRI nor scMRI techniques directly measure anatomic connectedness. Moreover, direct associations between functional synchrony and underlying anatomic structure have yet to be established in autism.

Our whole-brain seed-based approach differs from most neuroimaging studies examining pairwise correlations between *a priori* ROIs. Such studies limit resolution of network-level effects and allow limited conclusions regarding whole-brain or whole-network abnormalities. Whole-brain or whole-network fcMRI approaches indeed report both under- and overconnectivity, particularly within the DMN [25], [27], [45], [69], [78]. In addition, our method is not biased by Euclidean distance between presumably connected neural substrates, as local and long-range structural covariance is captured equally by our analyses. Our results suggest decreased local and long-range connectivity in the salience network, as well as altered (both increased and decreased) local and long-range connectivity within the DMN. The fundamental causes of brain dysfunction and altered structure in autism may not reflect a global insult or gross morphological change, but may instead emerge via selective processes resulting in abnormal architecture of specific neural networks underlying the clinical manifestations of the disease.

Our data suggest that discrepancies in fcMRI over- and under-connectivity, WM and GM volume, cortical thickness, and WM integrity measures may be reconciled by a model of autism as a network-based disease. Recent fcMRI studies are consistent with the concept of autism as a disease with distinctive network distribution patterns [38], [78]. Abnormal network architecture may be evident from early stages in the disease, and may reflect genetic, molecular, or cellular neuropathology in specific networks or nodes [77]. Whether these network alterations are permissive or reactive remains unknown. The age-dependent topology of large-scale structural networks in childhood has recently been identified [58], but the interrelationships of various structural and functional network-level measures have yet to be elucidated. Age-related patterns within these networks remain understudied in autism, but future work may reveal network- or node-specific abnormalities in genetic, molecular, or microstructural development in critical periods throughout development as well as later stages of the disease. More work in younger children is needed to clarify whether early disruptions may result in emergence of stable abnormal network architecture, further impacting network operation and cross-network interrelationships. Moreover, early overgrowth and later decline [2], [38], [79] may not be homogenous throughout the brain; rather, network-specific growth trajectories may contribute to regional and whole-brain over- and underdevelopment across ages. Larger cross-sectional and longitudinal studies of network development in autism are necessary to further clarify distinct network trajectory patterns.

These results could guide future studies of network abnormalities in autism. For example, our findings predict decreased fcMRI and WM connectivity between SN nodes, as well as increased connectivity between specific posterior elements within as well as outside of the canonical DMN. Moreover, it remains unknown whether other large-scale networks show similar patterns of abnormal structural architecture. Distinctive frontal, temporal, and cingulate gray matter overgrowth in young autistics, with relative sparing of occipital cortices has been reported [8], suggesting involvement of other networks beyond SN and DMN. In addition, whether distinct network topologies could characterize autism subtypes remains to be studied. Future scMRI investigations of other large-scale function-critical brain networks, such as those involved in executive function, speech and language, semantics, and primary sensorimotor functions, may reveal abnormal structural architecture to be a fundamental characteristic of network neurobiology in autism.

### Conclusions

Our study supports a model of autistic pathophysiology affecting domain-specific large-scale brain networks. Using standard anatomic MRI, we identified network-level structural abnormalities in the autistic brain, providing the first account of whole-brain, network-specific perturbations within autistic brain architecture. Our results suggest that structural brain abnormalities in autism may affect distinct large-scale networks. The SN appears underdeveloped in volume and extent, whereas the DMN demonstrates elements of both under- as well as over-development. These network-level perturbations are consistent with the clinical manifestations of the disease, and may provide targets for further study and intervention. Moreover, FI may represent an epicenter of perturbed structure and function in the autistic brain. Our work provides a unifying model of previously discordant findings based on structural and functional assessment, reconciles recent work with classic gross morphological findings in the disease, and reveals divergent network-dependent over- and underdevelopment in the same subjects. The diffuse specificity of our findings is consistent with emerging literature identifying regional abnormalities using varying techniques on microstructural as well as macrostructural levels.

## Supporting Information

Figure S1 **Structural covariance maps of the salience network in autism and controls, accounting for age.** Statistical parametric maps depict brain regions in which gray matter intensity covaried with that of the seed ROI (right FI) in each group. (A) Entering age as a covariate of no interest in the statistical model (yellow) had minimal affect on structural covariance topology in autistic subjects (blue; see also Table S2). (B) Corresponding scMRI map in normal controls (yellow, accounting for age; blue, as reported in the main body of the manuscript). scMRI data are T-statistic maps (p<0.01, FWE-corrected) displayed on the average anatomical template of all subjects. The left side of the image corresponds to the right side of the brain. FI, frontoinsula; FWE, family-wise error; ROI, region of interest; scMRI, structural covariance MRI; SN, salience network.
(TIF)

Table S1 Group age distributions.
(PDF)

Table S2 MNI coordinates and characteristics of peak voxels and associated clusters of groupwise scMRI maps, with age as a covariate in the model.
(PDF)

Table S3 MNI coordinates and characteristics of peak voxels and associated clusters of between-group scMRI maps, with age as a covariate in the model.
(PDF)
