# Supplementary figures and images for: scMRI Reveals Large-Scale Brain Network Abnormalities in Autism
Source: PLoS One. 2012 Nov 21;7(11):e49172. doi: 10.1371/journal.pone.0049172 (PMC3504046; doi:10.1371/journal.pone.0049172)

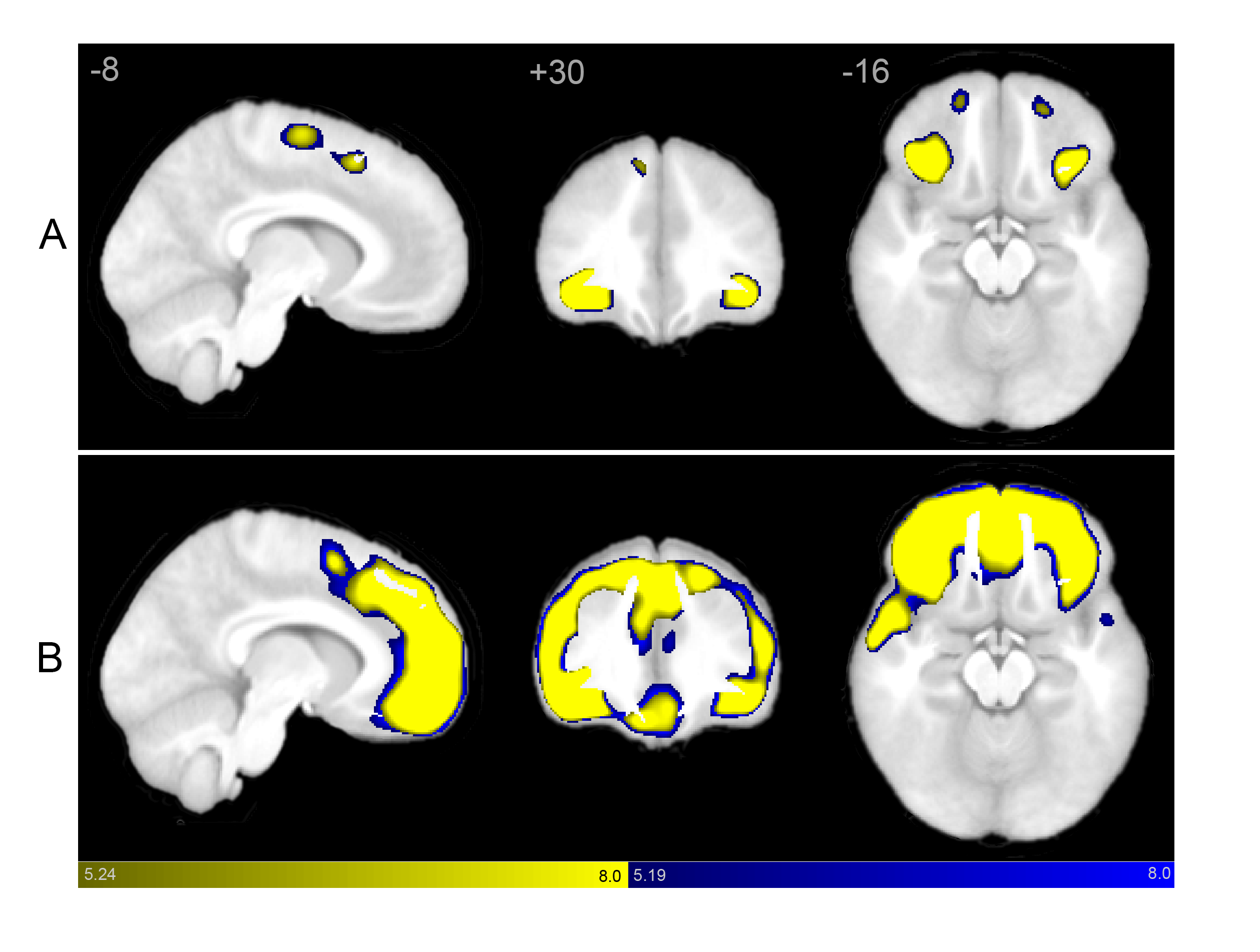

Supplement: Figure S1 — Structural covariance maps of the salience network in autism and controls, accounting for age. Statistical parametric maps depict brain regions in which gray matter intensity covaried with that of the seed ROI (right FI) in each group. (A) Entering age as a covariate of no interest in the statistical model (yellow) had minimal affect on structural covariance topology in autistic subjects (blue; see also Table S2). (B) Corresponding scMRI map in normal controls (yellow, accounting for age; blue, as reported in the main body of the manuscript). scMRI data are T-statistic maps (p<0.01, FWE-corrected) displayed on the average anatomical template of all subjects. The left side of the image corresponds to the right side of the brain. FI, frontoinsula; FWE, family-wise error; ROI, region of interest; scMRI, structural covariance MRI; SN, salience network. (TIF) [file pone.0049172.s001.tif]
