# Supplementary material for: scMRI Reveals Large-Scale Brain Network Abnormalities in Autism
Source: PLoS One. 2012 Nov 21;7(11):e49172. doi: 10.1371/journal.pone.0049172 (PMC3504046; doi:10.1371/journal.pone.0049172)
Supplement: Table S1 — Group age distributions. (PDF) [file pone.0049172.s002.pdf]

	Mean Age (yrs.)	S.D. Age (yrs)	Age Range (yrs.)	3 - 7 yrs (n)	7 .1 - 11 yrs	11.1 - 15 yrs	15.1 - 19 yrs	19.1 - 23 yrs
autism	13.27 (5.07)	5.07	3.49 - 22.33	6	11	13	11	8
control	13.67 (5.53)	5.53	3.47 - 22.44	6	11	13	7	12

Table S1. Group age distributions.
