# Supplementary material for: scMRI Reveals Large-Scale Brain Network Abnormalities in Autism
Source: PLoS One. 2012 Nov 21;7(11):e49172. doi: 10.1371/journal.pone.0049172 (PMC3504046; doi:10.1371/journal.pone.0049172)
Supplement: Table S2 — MNI coordinates and characteristics of peak voxels and associated clusters of groupwise scMRI maps, with age as a covariate in the model. (PDF) [file pone.0049172.s003.pdf]

	x	y	z	p (FWE)	T height	Peak Region	Secondary Regions	
**SN (R FI seed)**								
autism	38	26	-10	0.000	93.27	R FI	R Insula	
	-37	27	-13	0.000	8.81	L FI	L Insula	L Temporal Pole
	-30	21	-19	0.000	6.32	L FI	L Insula	L Temporal Pole
	10	-6	49	0.000	6.31	R SMA	R Mid Cingulum	R Sup Frontal
	10	13	50	0.002	5.6	R SMA	R Sup Frontal	R Mid Cingulum
	9	27	49	0.002	5.67	Medial R Sup Frontal	R SMA	R Sup Frontal
	-12	-9	60	0.000	6.2	L SMA	L Precentral	L Sup Frontal
	-7	16	48	0.001	5.99	L SMA	L Sup Frontal	L Mid Cingulum
	-20	8	55	0.002	5.71	L Sup Frontal	L Mid Frontal	
	-17	16	53	0.003	5.55	L Sup Frontal	L Mid Frontal	
	-21	48	-15	0.003	5.51	L Mid Frontal Orb	L Sup Frontal Orb	L Inf Frontal Orb
	18	53	-17	0.004	5.45	R Sup Frontal Orb	R Mid Frontal Orb	
	20	56	1	0.009	5.24	R Sup Frontal	R Sup Frontal Orb	R Med Sup Frontal
	16	4	60	0.009	5.24	R SMA	R Sup Frontal	
control	38	26	-10	0.000	109.52	R FI	R Insula	
	23	59	2	0.000	10.99	R Sup Frontal	R Sup Frontal Orb	
	-24	58	-3	0.000	10.15	L Sup Frontal Orb	L Sup Frontal	
	-49	-12	-34	0.000	6.13	L Inf Temporal	L Mid Temporal	
	10	-10	49	0.002	5.6	R SMA	R Mid Cingulum	
	-37	-26	-27	0.003	5.54	L Fusiform	L Inf Temporal	
	-36	7	47	0.003	5.52	L Mid Frontal	L Precentral	
	7	4	55	0.006	5.37	R SMA	L SMA	
**DMN (R PCC seed)**								
autism								
	4	-40	36	0.000	283.11	R Mid Cingulum	L Mid Cingulum	B Precuneus
	11	-70	42	0.000	8.54	R Precuneus	R Cuneus	
	57	-51	14	0.000	7.09	R Mid Temporal	R Sup Temporal	
	-34	-76	36	0.000	6.94	L Mid Occipital	L Inf Parietal	
	-2	-16	-19	0.000	7.42	L Ventral Tegmental		
	-5	-29	12	0.000	7.06	L Thalamus		
	-13	-25	-22	0.000	6.82	L Parahippo		
	0	-26	14	0.000	7.11	L Thalamus		
	-44	-77	20	0.000	6.32	L Mid Occipital	L Mid Temporal	L Angular
	-30	-56	52	0.003	5.52	L Inf Parietal	L Sup Parietal	
	-54	11	-14	0.000	6.62	L Sup Temporal Pole		
	57	-23	-4	0.000	6.19	R Sup Temporal	R Mid Temporal	
	61	-10	-1	0.005	5.44	R Sup Temporal	R Heschl	
	-59	-2	9	0.001	5.89	L Rolandic Oper	L Postcentral	L Heschl
	-15	-23	-31	0.001	5.83	L Parahippo	L Fusiform	
	46	-14	0	0.001	5.73	R Sup Temporal	R Insula	R Heschl
	-20	15	-24	0.003	5.52	L Inf Frontal Orb	L Sup Frontal Orb	
	9	-21	72	0.004	5.49	R Paracentral Lobule	R SMA	R Precentral
	11	-21	-14	0.004	5.49	R Parahippo		
	26	12	-32	0.006	5.37	R Sup Temporal Pole	R Parahippo	
	-17	-29	23	0.006	5.36	L Caudate		
	19	-24	-33	0.006	5.35	R Cerebellum 3	R Cerebellum 4,5	R Parahippo
	-16	-11	-32	0.006	5.35	L Parahippo	L Hippocampus	L Fusiform
	40	-17	13	0.009	5.25	R Insula	R Rolandic Oper	R Heschl
	-10	-74	42	0.009	5.24	L Precuneus	L Sup Parietal	
	-22	-90	-22	0.009	5.23	L Cerebellum Crus	L Lingual	L Fusiform
control	4	-40	36	0.000	261.61	R Mid Cingulum	L Mid Cingulum	B Precuneus
	-9	-52	64	0.000	6.97	L Precuneus	L Mid Cingulum	
	38	-55	51	0.000	6.53	R Inf Parietal		
	-18	54	37	0.003	5.58	L Sup Frontal	R Sup Parietal	
	7	-50	2	0.000	6.09	Vermis 4,5	R Lingual	R Precuneus
	-57	-47	35	0.000	6.68	L Supramarginal	L Inf Parietal	L Angular
	-56	-30	42	0.000	6.63	L Inf Parietal	L Supramarginal	
	-62	-9	32	0.002	5.63	L postcentral	L Precentral	
	62	2	25	0.000	6.53	R Precentral	R Postcentral	
	52	-50	42	0.001	5.99	R Inf Parietal	R Supramarginal	R Angular
	56	-51	14	0.000	6.49	R Mid Temporal	R Sup Temporal	
	-27	-56	54	0.000	6.4	L Sup Parietal	L Inf Parietal	
	-57	-54	-5	0.001	5.76	L Mid Temporal	L Inf Temporal	
	14	59	34	0.003	5.51	R Sup Frontal		R Angular
	-27	14	61	0.004	5.44	L Mid Frontal	L Sup Frontal	
	-22	-38	66	0.005	5.44	L Postcentral	L Sup Parietal	

Table S2. MNI coordinates and characteristics of peak voxels and associated clusters of groupwise scMRI maps, with age as a covariate in the model.
FWE, family-wise error.
