# Supplementary material for: scMRI Reveals Large-Scale Brain Network Abnormalities in Autism
Source: PLoS One. 2012 Nov 21;7(11):e49172. doi: 10.1371/journal.pone.0049172 (PMC3504046; doi:10.1371/journal.pone.0049172)
Supplement: Table S3 — MNI coordinates and characteristics of peak voxels and associated clusters of between-group scMRI maps, with age as a covariate in the model. (PDF) [file pone.0049172.s004.pdf]

	x	y	z	p (UC)	T height	Peak region	Secondary Regions		
**SN (R FI seed)**									
autism > control	-11	-10	60	0.014	2.23	L SMA	L Paracentral Lobule	L Precentral	
	36	27	-10	0.020	2.08	R Inf Fronal Orb (FI)	R Insula		
	12	-3	49	0.023	2.02	R SMA	R Mid Cingulum		
	-19	7	55	0.031	1.88	L Sup Frontal	L Mid Frontal		
	24	29	-9	0.041	1.75	R Caudate	R Putamen	R Insula	
	-29	23	-16	0.048	1.67	L Inf Frontal Orb	L insula		
control > autism	42	39	-14	0.000	4.24	R Mid Frontal Orb	R Inf Frontal Orb (FI)		
	27	55	12	0.000	3.96	R Sup Frontal	R Mid Frontal		
	42	37	23	0.000	3.89	R Mid Frontal	R Inf Frontal Tri		
	-8	51	14	0.000	3.61	L Medial Sup Frontal	L Ant Cingulate		
	-7	48	23	0.001	3.13	L Medial Sup Frontal	L Sup Frontal	L Ant Cingulate	
	-5	31	38	0.002	2.96	L Medial Sup Frontal	L Mid Cingulate		
	46	8	-31	0.001	3.26	R Mid Temporal Pole	R Sup Temporal Pole		
	-31	44	25	0.001	3.06	L Mid Frontal	L Sup Frontal		
	56	11	6	0.003	2.81	R Inf Frontal Oper	R Precentral		
	49	18	31	0.006	2.53	R Inf Frontal Oper	R Inf Frontal Tri		
	16	53	19	0.036	1.81	R Sup Frontal	R Medial Sup Frontal		
	-49	29	12	0.031	1.88	L Inf Frontal Tri			
	55	12	17	0.035	1.83	R Precentral	R Rolandic Oper		
	-46	33	4	0.046	1.69	L Inf Frontal Tri	L Inf Frontal Orb (FI)		
	36	31	42	0.048	1.67	R Mid Frontal			
**DMN (R PCC seed)**									
autism > control	14	-73	40	0.002	2.92	R Precuneus	R cuneus		
	30	-71	42	0.018	2.12	R Sup Occipital	R Angular	R Mid Occipital	
	2	-70	30	0.019	2.09	R Precuneus	L cuneus	L Precuneus	R cuneus
	-32	-77	35	0.009	2.39	L Mid occipital	L Inf parietal		
	-13	-26	-21	0.011	2.33	L Parahippo	L Cerebellum 3,4,5	L Fusiform	
	43	-76	28	0.018	2.13	R Mid Occipital	R Angular		
	-54	9	-12	0.018	2.12	L Sup Temporal Pole	L Sup Temporal	L Mid Temporal	
	53	-64	24	0.030	1.9	R Angular	R Mid Temporal	R Mid Occipital	
	58	-24	-3	0.030	1.9	R Sup Temporal	R Mid Temporal		
	59	-53	-13	0.011	2.33	R Inf Temporal	R Mid Temporal		
	57	-57	-2	0.046	1.69	R Mid Temporal	R Inf temporal		
	-47	-73	19	0.031	1.89	L Mid Occipital	L Mid Temporal		
	8	-37	-1	0.036	1.82	R Lingual	R Precuneus	R Thalamus	
	1	-28	-4	0.041	1.74	R Cerebellum 3,4,5	R Vermis 3		
	48	-13	1	0.044	1.71	R Sup Temporal	R Insula	R Heschl	
control > autism	-57	-45	37	0.003	2.77	L Inf Parietal	L Supramarginal		
	61	2	23	0.005	2.67	R precentral	R postecentral		
	45	-38	53	0.029	1.91	R Inf Parietal	R postcentral		
	59	-27	39	0.030	1.89	R postcentral	R Supramarginal		
	-7	-57	57	0.007	2.52	L precuneus			
	0	-49	-4	0.018	2.11	Vermis 3,4,5	L Cerebellum 3		
	-21	-57	55	0.019	2.1	L Sup Parietal	L precuneus	L Inf Parietal	
	-56	-54	-3	0.024	2.01	L mid temporal	L Inf temporal		
	63	-19	19	0.031	1.89	R Supramarginal	R Rolandic Oper	R Postcentral	R Sup Temporal
	-62	-8	32	0.031	1.89	L postcentral	L Precentral		
	-28	12	62	0.041	1.74	L Mid Frontal	L Sup Frontal		
	40	-59	54	0.044	1.71	R Sup Parietal	R Inf Parietal		
	-29	14	60	0.045	1.7	L Mid Frontal	L Sup Frontal		
	17	-42	70	0.048	1.67	R Postcentral	R Paracentral Lobule		

Table S3. MNI coordinates and characteristics of peak voxels and associated clusters of between-group scMRI maps, with age as a covariate in the model.
UC, uncorrected.
